# Chronic Lead Toxicity Presenting With Neurologic Decline in an Adult Male

**DOI:** 10.7759/cureus.106944

**Published:** 2026-04-13

**Authors:** Audrey Schulze, Vasilis Mavratsas, Patrick Sweet

**Affiliations:** 1 Internal Medicine, School of Medicine, University of Texas Medical Branch at Galveston, Galveston, USA; 2 Internal Medicine and Aerospace Medicine, University of Texas Medical Branch at Galveston, Galveston, USA; 3 Internal Medicine, University of Texas Medical Branch at Galveston, Galveston, USA

**Keywords:** blood lead level, environmental toxicology, gait instability, heavy metal exposure, lead toxicity, neurocognitive decline

## Abstract

Lead toxicity in adults is uncommon and can present with progressive neurological manifestations that mimic neurodegenerative diseases. We describe the case of a 61-year-old man with a several-year history of worsening gait instability, cognitive decline, dysarthria, and involuntary movements in the setting of persistently elevated blood lead levels. The patient’s symptoms began in late 2023 with balance difficulties and frequent falls, followed by progressive cognitive impairment, personality changes, and a 100 lb unintentional weight loss. His clinical course was further complicated by chronic non-healing wounds and imaging evidence of renal and hepatic scarring. Extensive neurological evaluation, including CT and MRI of the brain and genetic testing for Huntington’s, was unrevealing. The patient was found to have repeatedly elevated blood lead levels, initially in the 30 μg/dL range and later peaking in the 60 μg/dL range, despite cessation of work at the chemical and metal processing facility he had been employed at. Evaluation of potential ongoing sources revealed multiple retained BB fragments in the hand and foot dating back to early adulthood. These were initially considered as possible contributors, but point-of-care testing of the fragments on removal was negative for lead. A fragment in the foot was embedded in the bone and not amenable to removal. Additionally, possible residential exposure was considered, as the patient resided in a mid-20th-century home. He required multiple hospitalizations for progressive neurologic decline and elevated lead levels, during which he received inpatient chelation therapy with succimer. Chelation was associated with partial improvement and modest cognitive stabilization, though his dysarthria and gait ataxia persisted. His outpatient course was complicated by financial barriers that limited sustained chelation therapy, resulting in recurrent elevations of blood lead levels. Ongoing management included serial monitoring of lead levels following chelation, with plans to pursue surgical removal of a retained abdominal wall wire if levels were to rise again, suggesting an endogenous source.

## Introduction

Lead toxicity is an increasingly rare but clinically significant diagnosis in adults in developed countries, where environmental regulations have substantially reduced population-level exposure [1]. However, when lead toxicity presents, it can manifest insidiously and mimic neurodegenerative or movement disorders, leading to potential diagnostic delay. In the United States, fewer than 1% of adults have blood lead levels greater than 10 μg/dL, and symptomatic toxicity is uncommon, particularly outside of known occupational exposures [2,3]. Despite this rarity, even moderate elevations in blood lead levels have been associated with meaningful neurological and systemic morbidity, underscoring the importance of maintaining clinical suspicion in atypical presentations. Understanding how lead produces these clinical effects is critical to recognizing its often subtle and multisystem presentation.

Lead exerts multisystem toxicity through well-established mechanisms affecting the nervous system, kidneys, and cardiovascular system. Neurologically, lead disrupts calcium-dependent neuronal signaling, impairs neurotransmitter release, induces oxidative stress, and promotes neuroinflammation, resulting in cognitive dysfunction, behavioral changes, and motor impairment [4,5]. Adult exposure has been associated with deficits in executive function, memory, and processing speed, as well as motor impairment, including gait instability and incoordination. These effects can persist despite the removal of the exposure. Renal toxicity is characterized by chronic tubulointerstitial injury, evidenced by population-based analyses from the National Health and Nutrition Examination Survey (NHANES), which demonstrated an association between blood lead concentrations as low as approximately 2.4 µg/dL and reduced estimated glomerular filtration rate in adults [6]. Cardiovascular effects include hypertension and increased cardiovascular risk, thought to be contributed from dysfunction of endothelial cells, oxidative stress, and impaired bioavailability of nitric oxide [7].

Although these effects of lead on the body are scientifically recognized, several gaps remain in the understanding of lead toxicity in adults. Long-term neurological outcomes following chelation therapy are poorly characterized, particularly with respect to the reversibility of motor versus cognitive dysfunction [1]. Financial and systemic barriers to long-term chelation therapy may further limit treatment effectiveness and contribute to incomplete recovery, yet these real-world constraints are infrequently addressed in the literature.

We report a case of severe, progressive, neurologic decline associated with chronic lead toxicity that mimicked a neurodegenerative disorder, involved the potential of multi-source exposure, and demonstrated partial yet incomplete recovery following chelation, highlighting critical diagnostic and therapeutic challenges.

## Case presentation

Clinical findings

A 61-year-old man presented with a several‑year history of progressive neurologic decline. Initial symptoms began in late 2023 with gait instability and frequent falls, followed by worsening dysarthria, involuntary movements, and cognitive impairment characterized by memory loss, personality changes, and diminished executive function. Over approximately one year, he experienced unintentional weight loss of nearly 100 pounds. His course was complicated by chronic non‑healing wounds and progressive functional decline requiring repeated hospitalizations. There was no family history of neurodegenerative disease. Neurologic examination revealed marked gait instability, dysarthric speech, and abnormal involuntary movements. Cognitive testing demonstrated global impairment without a focal cortical pattern.

Investigations

Initial neuroimaging with CT and MRI of the brain showed no acute intracranial pathology or structural abnormalities to explain his symptoms. Genetic testing for Huntington’s disease was negative. Routine laboratory evaluation revealed persistently elevated blood lead levels, initially in the 30 µg/dL range and later rising into the 60s µg/dL (normal blood lead levels are <3.5 µg/dL). The patient was also found to have normocytic anemia throughout his disease course.

**Figure 1:**
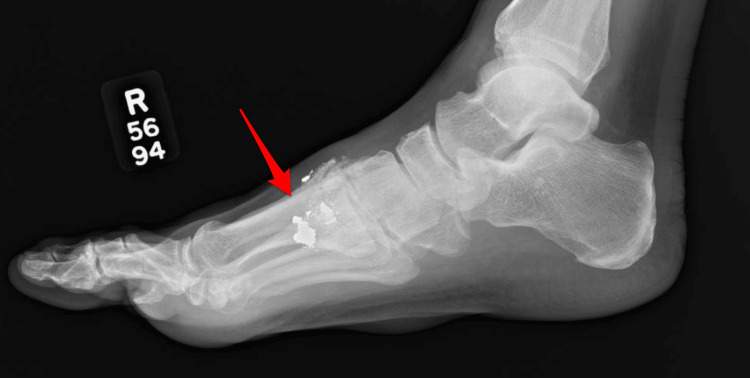
Right foot X-ray with embedded BB pellet fragments.

**Figure 2:**
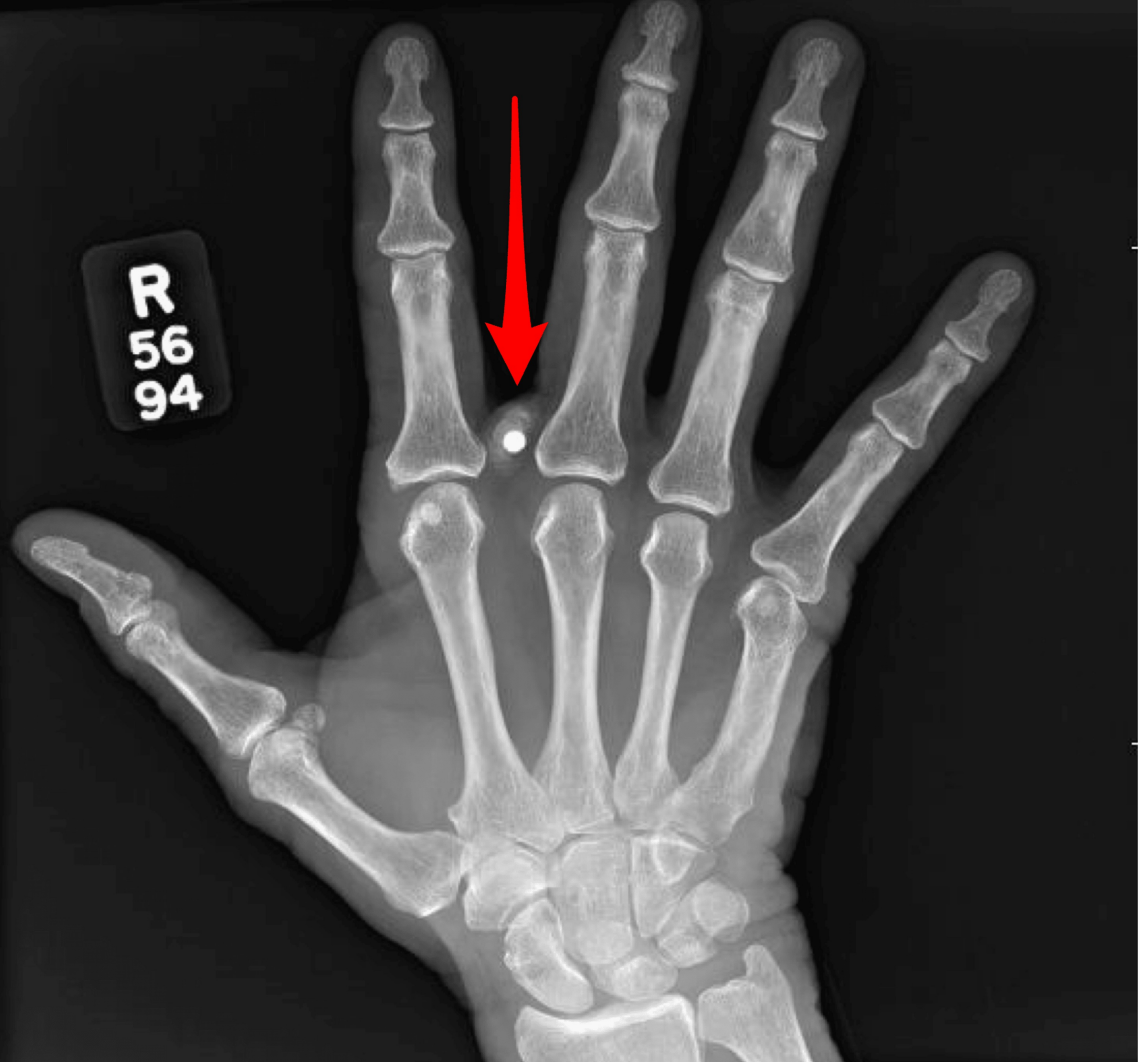
Right hand X-ray with BB pellet before removal.

**Figure 3:**
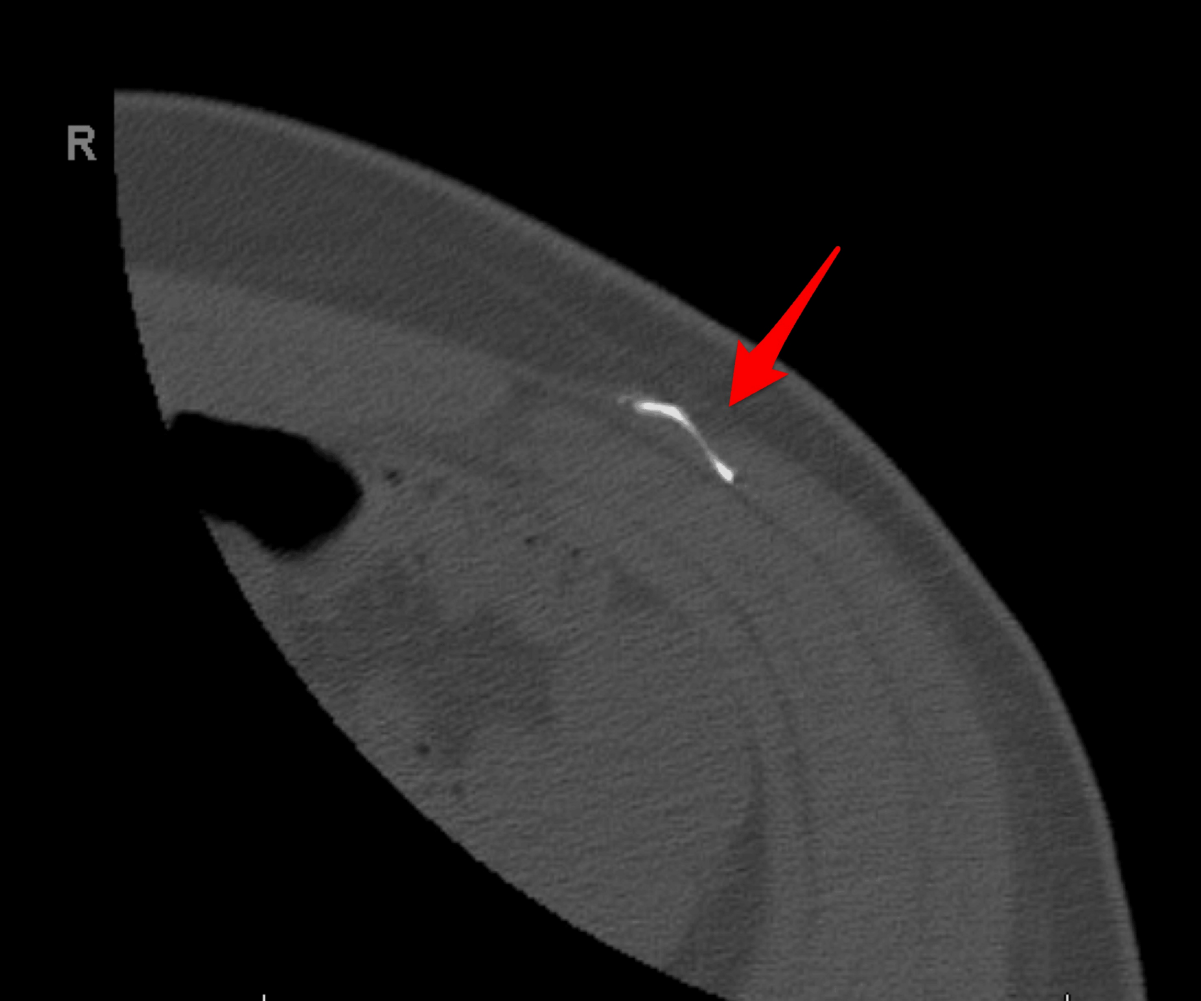
Metal wire in the upper left abdominal wall found on CT scan.

Despite cessation of suspected occupational exposure at a chemical and metal processing facility, lead levels remained elevated. Further evaluation identified retained BB fragments in the right hand and right foot from remote injuries earlier in life (Figures 1, 2). BB pellets are small, spherical projectiles typically made of steel or lead, designed to be fired from air-powered guns, commonly referred to as BB guns. The BB from the hand was removed, and point‑of‑care testing was negative for lead. The fragment in the foot was fully embedded within bone and not amenable to removal. A retained abdominal wall wire fragment was also identified and evaluated by general surgery (Figure 3). At the time, its contribution to lead exposure had not been established, and conservative management with surveillance was recommended. These are potential sources of ongoing endogenous exposure. Possible residential exposure was considered, as the patient lived in a mid‑20th‑century home.

Differential diagnosis

The differential diagnosis included neurodegenerative disease (including Huntington’s disease), atypical Parkinsonism, autoimmune or paraneoplastic encephalopathy, metabolic or toxic encephalopathy, and chronic heavy metal toxicity. Given the persistently elevated blood lead levels, partial biochemical response to chelation, and exclusion of primary neurodegenerative causes, chronic lead toxicity was felt to best explain the clinical picture.

Treatment

The patient underwent multiple hospitalizations for symptomatic lead toxicity and received inpatient chelation therapy with succimer. Chelation resulted in transient reductions in blood lead levels and modest stabilization of cognitive symptoms. Long‑term outpatient chelation was limited by financial barriers. Management focused on serial monitoring of blood lead levels following chelation, with a plan to surgically remove the retained abdominal wall wire fragment if lead levels rose again, suggesting an endogenous source.

Outcome and follow-up

Following chelation therapy, blood lead levels decreased, though gait instability and dysarthria persisted. Cognitive decline stabilized but did not fully reverse. The patient continued to require close outpatient follow‑up with serial lead level monitoring. At the last follow‑up, further intervention was contingent on biochemical trends, with surgical removal of the retained abdominal fragment under consideration if recurrent elevations occurred.

## Discussion

Causes of lead poisoning in adults

In adults, lead toxicity commonly occurs as a result of occupational exposure, retained lead-containing foreign bodies, or environmental sources such as older housing. In this case, the patient’s occupational exposure was at a chemical and metal processing facility. Occupational lead exposure remains the leading cause of adult lead toxicity in developed countries, particularly in industries involving metal processing, manufacturing, or recycling, and may result in substantial cumulative burden even after exposure ends [8].

The persistence and rise in blood lead levels after removal from the occupational exposure suggest an endogenous source rather than continued exogenous exposure. Overall, 95% of the total body lead burden in adults is stored in bone, where it can remain for decades and slowly redistribute into the bloodstream, especially during physiologic stress, illness, or weight loss [2]. Retained BB and wire fragments represent an additional potential endogenous source. Although the BB fragments tested negative for lead in this patient, the presence of a retained abdominal wall wire fragment raised concern for intermittent or delayed leaching. Surgical removal of retained fragments has been shown to reduce ongoing lead exposure when chelation alone is insufficient, though feasibility depends on fragment location and surgical risk [9]. Environmental exposure from a mid-20th-century home was also considered, as lead-based paint and contaminated dust remain important contributors to chronic exposure in those living in older homes [10]. The existence of multiple potential sources complicates attribution and management, a common challenge in chronic adult lead toxicity.

Pathophysiology and clinical features

Lead is neurotoxic through a variety of mechanisms, including the disruption of calcium-dependent neuronal signaling, inhibition of neurotransmitter release, oxidative stress, mitochondrial dysfunction, and neuroinflammation [11]. These processes often affect the cerebellum, basal ganglia, and prefrontal cortex, providing a basis for the patient’s ataxia, dysarthria, and progressive cognitive and behavioral changes [12].

The gait ataxia and dysarthria experienced by this patient resulted from lead interfering with calcium channel function and synaptic plasticity within cerebellar and motor pathways [12]. Lead-induced dysfunction in glutamate and GABA neurotransmission impaired precise motor control and speech production [13]. In the cognitive domain, the patient’s memory loss, personality changes, and executive dysfunction reflect lead’s impact on the prefrontal cortex through NMDA receptor signaling, mitochondrial dysfunction, and oxidative stress [14]. The involuntary movements observed are consistent with the effect of lead on the basal ganglia and motor control pathways, where tremors and hyperkinetic features have been found in patients with chronic lead toxicity [15].

The patient’s anemia is a classic sign of lead toxicity, resulting from the inhibition of δ-aminolevulinic acid dehydratase and shortened erythrocyte lifespan due to oxidative damage to the membrane [16]. Chronic exposure to lead in adults can also lead to irreversible organ damage, including chronic tubulointerstitial nephritis, cardiovascular disease, and hepatotoxicity, among others [1,6,7,17].

Diagnosis

This case emphasizes the importance of occupational and environmental history. The combination of progressive neurologic decline, ataxia, and cognitive impairment in a middle-aged adult often suggests a neurodegenerative etiology. However, when neuroimaging is unremarkable, which is typical in uncomplicated lead poisoning, clinicians should broaden the differential to include toxic, metabolic, and nutritional etiologies [18,19].

Current guidelines suggest tailoring laboratory testing to a patient’s specific risk profile, but, in practice, this individualized approach can lead to inconsistent toxicology screening [20]. Standard workup for suspected dementia includes a complete blood count, metabolic panel, thyroid function, and vitamin B12, but heavy metal screening is an “optional” category only to be included if there is specific clinical suspicion [20]. The downside of this approach is that it relies on the clinician taking a comprehensive history and identifying the exposure during initial evaluation. This prolongation in diagnosis can lead to prolonged exposure to heavy metals and delayed treatment.

Blood lead measurement is a low-cost intervention that should be considered early in the workup of unexplained encephalopathy. Measuring free erythrocyte protoporphyrin or zinc protoporphyrin can be useful, as elevations over 35 μg/dL can help confirm chronic exposure [16]. This is because lead inhibits ferrochelatase in the heme synthesis pathway, causing protoporphyrin accumulation in erythrocytes.

Management and therapeutic considerations

Chelation therapy with succimer (dimercaptosuccinic acid, DMSA) is the preferred oral treatment for moderate lead toxicity in adults. Succimer binds circulating lead and enhances urinary excretion while limiting redistribution into the central nervous system [21]. During active therapy, blood lead levels may decrease substantially, though the response is highly variable. Rebound elevation following discontinuation is well described, with levels often returning to 60-85% of pretreatment values within weeks due to redistribution from skeletal stores [22].

This patient followed this expected pattern, with transient reductions in blood lead levels during inpatient chelation followed by rebound elevations when outpatient therapy was interrupted. The long biological half-life of lead in bone and its predominant storage in skeletal tissue help explain why single chelation courses are insufficient in cases of substantial cumulative exposure [22]. Repeated or prolonged courses are often necessary, particularly when endogenous sources persist. Chelation alone does not address retained lead-containing foreign bodies, and combination management with surgical removal is considered the optimal strategy when feasible [23].

Outcomes and clinical implications

The patient’s outcome revealed a divergence in recovery patterns. Cognitive stabilization following chelation suggested that synaptic interference in the prefrontal cortex was partially reversible once the toxic burden was reduced. Conversely, the persistence of gait ataxia and dysarthria implies that the structural damage to the cerebellar motor pathways was chronic and potentially irreversible [19]. This supports the observation that encephalopathic symptoms may persist after chelation and that the degree of reversibility is inversely correlated with the duration and severity of exposure [24].

This case reinforces several critical clinical learning points. Lead toxicity, though less common than in previous decades, should remain in the differential diagnosis for adults presenting with progressive neurological decline, especially when features span across multiple domains such as cognitive, motor, and systemic. Multisource exposure complicates diagnosis and management, and diagnostic delay is common due to the presentation mimicking other conditions. Real-world barriers to prolonged chelation therapy may limit neurologic recovery even after correct diagnosis, emphasizing the need for realistic patient expectations and longitudinal monitoring [23]. This case exemplifies how even appropriately diagnosed and treated lead toxicity can result in incomplete recovery and residual disability, highlighting the importance of early recognition and sustained access to treatment.

## Conclusions

This case highlights the diagnostic challenges posed by chronic lead toxicity in adults presenting with progressive neurologic decline. The combination of gait ataxia, dysarthria, cognitive impairment, and systemic involvement illustrates lead’s capacity to mimic primary neurodegenerative disease and contribute to prolonged diagnostic delay. Although succimer-based chelation therapy resulted in moderate improvement and stabilization of cognitive symptoms, persistent motor dysfunction and rebound elevations in blood lead levels highlight the limitations of pharmacologic treatment without definitive source control. This case also highlights practical barriers to treatment that patients may face, such as financial constraints. Clinicians should recognize that incomplete neurologic recovery is common in severe chronic exposure and that optimal management includes close follow-up to monitor symptoms.
